# Is Vaginal Laxity Associated with Vaginal Parity and Mode of Delivery?

**DOI:** 10.1007/s00192-024-05849-6

**Published:** 2024-07-13

**Authors:** Susana Mustafa-Mikhail, Moshe Gillor, Yara Nakhleh Francis, Hans Peter Dietz

**Affiliations:** 1 Sydney Medical School Nepean, University of Sydney, Sydney, NSW Australia; 2 Galilee Medical Center, Naharyia, Affiliated to The Azrieli Faculty of Medicine of Bar-Ilan University, Yermiaho Halperin 2, Haifa, Safed Israel; 3 Kaplan Medical Center, Affiliated to the Hebrew University and Hadassah School of Medicine in Jerusalem, Rehovot, Israel; 4 Sydney Urodynamic Centres, Penrith, NSW Australia

**Keywords:** Vaginal laxity, Hiatal area, Parity, Translabial ultrasound

## Abstract

**Introduction and Hypothesis:**

Vaginal laxity (VL) is a common symptom of pelvic floor dysfunction. Although VL has become a frequent topic for research in the last decade, its pathogenesis is still not well understood. The objective was to determine whether vaginal parity or mode of delivery is associated with vaginal laxity.

**Methods:**

This was a retrospective observational study involving women seen in a tertiary urogynecology clinic between May 2016 and November 2018 with symptoms of pelvic floor dysfunction. Patients underwent a standardized interview, clinical examination (POP-Q), and four-dimensional (4D) pelvic floor ultrasound (PFUS). Data regarding vaginal parity and the mode of delivery were based on patient-reported information. Archived 4D-PFUS volumes were analyzed offline to evaluate levator hiatal area on Valsalva.

**Results:**

Data from 1,051 patients were analyzed. VL was reported by 236 women (23%) who were younger on average (mean age 54 vs 59 years, *p* < 0.001) and less likely to be menopausal (530 out of 815 [65.0%] vs.129 out of 236 [54.7%]), *p* = 0.004]. Symptoms of prolapse were much more common in the VL group (214 out of 236 [91%] vs 316 out of 815 [39%], *p* =  < 0.001) and on imaging mean levator hiatal area (HA) on Valsalva was larger (31 vs 26 cm^2^, *p* = 0.01). Vaginal parity was associated with VL symptoms (235 out of 236 [99%] vs 767 out of 815 [94%], *p* = 0.008), but neither VL prevalence nor bother increased with higher parity. Women who delivered vaginally were three times more likely to complain of VL than those who delivered only by cesarean section.

**Conclusions:**

Vaginal laxity was found to be more prevalent in vaginally parous women. This effect seems to be largely attributable to the first delivery. Instrumental delivery was not shown to increase association with VL compared with normal vaginal delivery.

## Introduction

Vaginal laxity (VL), which is defined as excessive vaginal looseness [1], has been increasingly reported by women with a wide range of bother. This symptom is often described to convey a negative effect on sexual relations and overall quality of life [2].

Vaginal laxity has long been under-reported by women and consequently under-diagnosed by their health physicians, as shown by a study demonstrating that 83% of urogynecologists felt that VL was under-reported by their patients [3]. The prevalence of VL among women attending a urogynecology clinic has been reported to be 24–38% [2, 4]. Previous studies have shown that VL is associated with bother as high as that described with pelvic organ prolapse (POP) symptoms [4]. VL was reported to be associated with younger age, vaginal parity, symptoms of POP, and objective prolapse [3, 4].

The etiology and risk factors of VL are not well established. However, strong associations were found with the sum of genital hiatus (gh) and perineal body (pb), measured on clinical examination according to Pelvic Organ Prolapse Quantification (POP-Q) system, as well as the levator hiatal area on Valsalva maneuver, measured on pelvic floor ultrasound (PFUS). This suggests that VL could be a manifestation of levator ani hyperdistensibility [4]. Given that the vaginal high-pressure zone is set by the puborectalis component of the levator ani muscle, injury to this muscle, which can subsequently cause an increase in the hiatal area, would be expected to result in a looser vagina [5]. Although some studies have shown that there is an association between levator ani injury, hyperdistensibility, and vaginal delivery, little is known about the effect of delivery mode on VL.

The aim of this study was to further investigate the relationship between VL and vaginal parity, as well as the mode of vaginal delivery.

## Materials and Methods

This is a retrospective observational study including all 1,078 women referred to a tertiary urogynecology clinic owing to symptoms of pelvic floor dysfunction, between May 2016 and November 2018. Patients underwent a standardized interview in which self-reported data about demographics, obstetric history, and symptoms were obtained. Patients were classified according to mode of delivery to Cesarean deliveries (CS; only Cesarean deliveries), vaginal delivery (VD; at least one vaginal delivery and no forceps delivery [FD]), FD (any forceps delivery and any vacuum delivery [no forceps]). VL was elicited by asking: “Have you noticed vaginal laxity or looseness?”, which is in fact component 4 of the validated ICIQ-VS questionnaire. The subjective bother of VL was quantified on a scale from 0 to 10 using a visual analogue scale (VAS) score, in those who gave a positive answer to the above question.

This was followed by a clinical examination using the POP-Q system on maximal Valsalva maneuver after bladder emptying. Clinically significant POP was defined as stage ≥ 2 in the anterior and/or posterior compartments, and/or ≥ 1 in the apical compartment [6].

A four-dimensional (4D) pelvic floor ultrasound (PFUS) was then performed with the patient in the supine position using a GE Voluson 730 Expert or Voluson S6 system (GE Medical Systems, Zipf, Austria) with an 8–4 MHz curved array volume transducer, as previously described [7]. Volume acquisition was performed at rest, on maximum Valsalva maneuver, and on maximum pelvic floor muscle contraction. Significant POP on PFUS was defined according to previously established cutoffs as either a bladder or rectal ampulla descent to ≥ 10 mm and ≥ 15 mm below the pubic symphysis respectively, or descent of the uterus to ≤ 15 mm above the pubic symphysis [8]. Assessment of levator integrity and diagnosis of levator avulsion on ultrasound was performed according to a previously described and standardized technique [9].

Stored 4D-PFUS volumes were analyzed at a later date using the proprietary software 4D View 10.5 (GE Medical Ultrasound Kretz GmbH, Zipf, Austria), blinded to all patient data. Levator hiatal area on maximum Valsalva was measured in the plane of minimal hiatal dimensions using the rendered volume technique [9]. A test–retest series of 20 consecutive cases for levator hiatal area on Valsalva was undertaken by two observers (the first and second co-authors). Good interobserver reliability of 0.743 was achieved as determined by intraclass correlation coefficient (ICC; single measures, absolute agreement definition).

Statistical analysis was performed using SAS version 9.4 (SAS Institute, Cary, NC, USA). Generalized linear modeling was used to compare the different groups using logistic regression. Multivariate models were adjusted for age and sexual activity. A *p* value < 0.05 was considered statistically significant. This retrospective study was approved by the Institutional Ethics Committee IRB approval (NBMLHD HREC ref. 13–70).

## Results

During the study period from May 2016 to November 2018, a total of 1,078 women were seen in a tertiary urogynecology service. Twenty-five women were excluded owing to missing information and 2 because of neuropathic bladder and myotonic dystrophy, leaving 1,051 women to whom the following data and analysis pertain.

The mean age was 57.6 (standard deviation [SD] 13.8) years, 660 (63%) were postmenopausal and their mean body mass index (BMI) was 29 (SD 6.3) kg/m^2^. A total of 317 (30%) had had a previous hysterectomy, 187 (18%) a previous POP repair, and 131 (13%) a previous anti-incontinence procedure. Parity was noted in the history of 1,002 women. Normal vaginal deliveries only were reported by 644 women, 61 had had only cesarean births, 268 had had at least one FD and 29 at least one vacuum delivery without forceps.

Mean age at first vaginal delivery was 24 (SD 5.18) years, mean birthweight at first vaginal delivery was 3,362 (SD 523) g. Most women presented with stress urinary incontinence (*n* = 763, 73%) and urgency urinary incontinence (*n* = 737, 70%), followed by obstructed defecation in 532 (51%), prolapse symptoms in 531 (50%), nocturia in 422 (40%) women, symptoms of voiding dysfunction in 370 (35%), increased daytime urinary frequency (defined as more than seven voids per day) in 303 (29%), and anal incontinence in 163 (16%).

Vaginal laxity was described by 236 (23%) women with a median bother of 6 (interquartile range [IQR] 3.6–8.85) and symptoms of POP were reported by 530 (50%) women with a median bother of 7 (IQR 3.9–8.85). Clinically, mean Ba, Bp, and C were −0.69 (SD 1.66) cm, −1 (SD 1.4) cm, and −4.34 (SD 3.16) cm respectively. Overall, 818 (78%) women had clinically significant POP as defined above, which was in the form of anterior compartment prolapse in 620 (76%), uterine prolapse in 280 (34%), vault prolapse in 81 (10%), and posterior compartment prolapse in 579 (71%). The mean sum of gh and pb was 7.7 (SD 1.6) cm.

On imaging, 697 women (66%) had sonographically significant POP involving the bladder, uterus, enterocele, and rectal ampulla in 419 (60%), 89 (13%) 72 (10%), and 439 (63%) respectively. Mean bladder and rectal ampulla descent were to 5.5 (SD 19.9) mm and 8 (SD 16.6) mm below the symphysis pubis respectively. Mean uterine descent (when apparent) was to −2.3 (SD 13.7) mm and mean enterocele descent (when apparent) was to 8 (SD 16.6) mm in relation to the symphysis pubis. The mean hiatal area on Valsalva was 27 (SD 9) cm^2^. Levator avulsion was diagnosed in 235 women (22%), and this was bilateral in 87 (37%). Women who were sexually active were significantly younger than those who were not (51 years vs 65 years, *p* < 0.001).

Comparison of women with VL with those without the symptom on univariate analysis showed no significant differences in BMI, neonatal birth weight at first vaginal delivery, previous hysterectomy, and previous anti-incontinence operations (Table 1). Conversely, patients reporting VL were younger (54 vs 59 years, *p* < 0.0001), were less likely to be menopausal (129 [54.7%] vs 530 [65.0%], *p* = 0.004) and more women in the VL group had undergone prolapse repair operations in the past (114 out of 814 [14%] vs 17 out of 236 [7%], *p* = 0.0063). Symptoms of POP were more common in the VL-positive group (214 out of 236 [91%] vs 316 out of 815 [39%], *p* ≤ 0.0001) and so was significant clinical POP (224 out of 235 [95%] vs 593 out of 813 [73%], *p* < 0.0001). Additionally, mean levator hiatal area on Valsalva was larger in VL-positive patients (31 vs 26 cm^2^, *p* = 0.01; Table 2).

**Table 1:** Demographics and patient history

	Vaginal laxity negative (*N* = 815)	Vaginal laxity positive (*N* = 236)	Odds ratio (95% CI)	*p* value
Age in years (mean ± SD)	58.5 ± 14.0	54.3 ± 12.3	0.98 (0.97–0.99)	< 0.001
BMI in kg/m2 (mean ± SD)	29.1 ± 6.1	29.3 ± 6.9	1.00 (0.98–1.02)	0.752
Menopause, *n* (%)	530 (65.0%)	129 (54.7%)	0.65 (0.48–0.87)	0.004
Vaginal parity, *n* (%)	767 (94.1%)	235 (99.6%)	14.71 (2.02–107.12)	0.008
Age at first delivery in years, mean ± SD	24.0 ± 5.0	24.9 ± 5.6	1.03 (1.00–1.06)	0.020
First neonatal birthweight (g), mean ± SD	3,352.1 ± 527.5	3,393.4 ± 547.5	1.00 (1.00–1.00)	0.312
Previous hysterectomy, *n* (%)	253 (31.1%)	63 (26.7%)	0.81 (0.58–1.12)	0.193
previous POP repair, *n* (%)	114 (14.0%)	17 (7.2%)	0.48 (0.28–0.81)	0.006
Previous anti-incontinence procedures, *n* (%)	139 (17.1%)	48 (20.3%)	1.24 (0.86–1.79)	0.246
Sexually active, *n* (%)	411 (50.4%)	142 (60.2%)	1.49 (1.11–1.99)	0.009

*CI* confidence interval, *SD* standard deviation, *BMI* body mass index, *POP* pelvic organ prolapse

**Table 2:** Clinical symptoms and findings on examination and ultrasound

	Vaginal laxity negative (*N* = 815)	Vaginal laxity positive (*N* = 236)	Odds ratio (95% CI)	*p* value
POP symptoms, *n* (%)	316 (46.5%)	214 (93.9%)	17.61 (10.05–30.87)	< 0.001
Any significant clinical POP, *n* (%)	593 (72.9%)	224 (95.3%)	7.56 (4.05–14.11)	**< **0.001
TVL in cm, mean ± SD	8.5 ± 1.1	8.4 ± 1.1	0.95 (0.83–1.09)	0.502
Ba, mean ± SD	−1 ± 1.6	0 ± 1.7	1.36 (1.25–1.48)	**< **0.001
C, mean ± SD	−5 ± 3.0	−3 ± 3.4	1.14 (1.09–1.19)	**< **0.001
Bp, mean ± SD	−1 ± 1.4	−1 ± 1.5	1.22 (1.10–1.34)	**< **0.001
gh + pb in cm, mean ± SD	7.5 ± 1.5	8.4 ± 1.5	1.45 (1.31–1.60)	**< **0.001
Mean Oxford, mean ± SD	2.3 ± 1.2	2.1 ± 1.1	0.84 (0.74–0.95)	0.007
Significant US POP, *n* (%)	497 (61.1%)	199 (84.7%)	3.52 (2.40—5.15)	**< **0.001
Bladder descent in mm, mean ± SD^a^	−2.4 ± 19.3	−15.8 ± 18.7	0.97 (0.96–0.97)	**< **0.001
Uterine descent in mm, mean ± SD^a^	−0.9 ± 10.6	−4.3 ± 14.3	0.98 (0.97–0.99)	**< **0.001
Rectal descent in mm, mean ± SD^a^	−2.1 ± 8.7	−2.4 ± 8.8	0.99 (0.98–1.01)	0.657
Enterocele descent in mm, mean ± SD^a^	−6.8 ± 17.8	−12.3 ± 15.3	0.98 (0.97–0.99)	**< **0.001
HA on Valsalva in cm^2^, mean ± SD	25.6 ± 8.6	31.3 ± 9.1	1.07 (1.05–1.09)	**< **0.001
Levator avulsion, *n* (%)	153 (18.8%)	81 (34.3%)	2.261 (1.640–3.117)	**< **0.001

*SD* standard deviation, *CI* confidence interval, *POP* pelvic organ prolapse, *TVL* total vaginal length, *HA* hiatal area

^a^A minus sign signifies a descent below the symphysis pubis, whereas a plus sign signifies an organ position above the symphysis pubis

Patients who reported VL were more likely to be sexually active (142 out of 236 [60%] vs 411 out of 815 [50%], *p* = 0.009), although this was insignificant on multivariate analysis.

Vaginal parity was associated with VL symptoms (231 out of 943 [25%] vs 5 out of 108 [5%], *p* < 0.0001); however, the latter did not increase in prevalence with increased vaginal parity and nor did the bother of this symptom (Table 3). Furthermore, in a separate analysis that included only vaginally parous patients, we did not observe any additional increase in VL symptoms with higher vaginal parity (Table 4).

**Table 3:** Vaginal parity, vaginal laxity (*VL*) symptoms and bother

	VD 0 (*n* = 108)	VD 1 (*n* = 130)	VD 2 (*n* = 346)	VD 3 (*n* = 292)	VD 4+ (*n* = 175)
VL symptoms	5 (5%)	28 (22%)	89 (26%)	76 (26%)	38 (22%)
OR^a^ (95% CI)	Ref. group	5.65 (2.101–15.219)	7.13 (2.82–18.1)	7.25 (2.85–18.46)	5.71 (2.17–15.02)
*p* value	**< **0.001				
Adjusted *p* value/OR^b^	**< **0.001	6.753 (2.480–18.393)	9.517 (3.693-24.523)	10.603 (4.069–27.631)	9.068 (3.354–24.514)
Mean VL bother (range)^c^	5.9 (2.1–10)	6.1 (1.3–10)	6.3 (1.6–10)	5.7 (0.7–10)	6.9 (1.9–10)

*OR* odds ratio, *VD* vaginal delivery

^a^All OR relates to comparison with vaginally nulliparous group

^b^Adjusted for age, sexually active state

^c^Only for patients with VL symptoms

**Table 4:** Sub-group analysis of only patients with vaginal delivery or deliveries

	VD 1 (*n* = 130)	VD 2 (*n* = 346)	VD 3 (*n* = 292)	VD 4+ (*n* = 175)
VL symptoms	28 (22%)	89 (26%)	76 (26%)	38 (22%)
OR (95% CI)	Ref. group	1.262 (0.78–2.04)	1.282 (0.78–2.1)	1.01 (0.58–1.75)
*p* value	0.5738			

*OR* odds ratio, *VD* vaginal delivery, *VL* vaginal laxity

Women who delivered vaginally were three times more likely to report VL than those who were nulliparous or delivered by CS only. Furthermore, those who had had at least one FD were four times more likely to report VL than those who were nulliparous or delivered by CS only (Table 5). There was a tendency toward greater association of instrumental delivery over normal vaginal delivery with VL; however, this did not reach statistical significance.

**Table 5:** Vaginal parity and delivery mode

	Nulliparous (*N* = 49)	Only CS (*N* = 61)	Only normal vaginal delivery/deliveries (*N* = 644)	At least one FD (*N* = 268)
VL symptoms	1 (2%)	5 (8%)	149 (23%)	72 (27%)
OR (95% CI)	Reference	4.286 (0.484–37.965)	14.448 (1.978–105.564)	17.633 (2.390–130.108)
	0.233 (0.026–2.067)	Reference	3.371 (1.326–8.570)	4.114 (1.585–10.679)
	0.069 (0.009–0.506)	0.297 (0.117–0.754)	Reference	1.220 (0.881–1.691)
	0.057 (0.008–0.418)	0.243 (0.094–0.631)	0.819 (0.591–1.136)	Reference
	0.046 (0.005–0.390)	0.198 (0.059–0.663)	0.669 (0.298–1.500)	0.816 (0.355–1.875)
*p* value	0.002			
Adjusted *p* value/OR^a^	**< **0.001	6.068 (0.676–54.487)	23.705 (3.183–176.547)	29.727 (3.944–224.057)
Mean VL bother (range)^b^	–	5.9 (2.1–10)	6.1 (1–10)	6.4 (0.7–10)

*CS* cesarean section, *VD* vaginal delivery, *VL* vaginal laxity, *FD* forceps delivery

^a^Adjusted for age, sexually active state

^b^Only for patients with VL symptoms (nulliparous = 0 [1 missing], CS = 4 [1 missing], FD = 56 [16 missing], NVD = 119 [30 missing])

## Discussion

In this study, VL was described by 23% of women attending our urogynecology unit, which is in accordance with previous reports [4]. It was found to be more prevalent in vaginally parous women. However, increased parity did not increase the likelihood of VL, or its bother. It seems that the association between vaginal childbirth and VL is largely attributable to the first delivery. This finding coincides with previous studies showing that most harmful effects on the pelvic floor are caused by the first vaginal delivery, with subsequent deliveries having less impact [10]. Van Gruting et al. evaluated the impact of first and second delivery on levator ani muscle (LAM) avulsion and its impact on pelvic floor dysfunction symptoms. The results showed that the first delivery carries the greatest risk for LAM avulsion. The second delivery could cause deterioration in LAM injury, but no new avulsion was seen [11].

As pelvic hyperdistensibility, manifested as a widened hiatal area, is more prevalent in women after vaginal and forceps delivery [12], this phenomenon, in our view, is likely to explain the increased rate of VL in vaginally parous women. There is growing evidence indicating the association between pelvic floor hyperdistensibility and VL. A publication by Manzini et al. [13] showed a statistically significant increase in sonographic measurements of pelvic floor distensibility in women with VL. Levator hiatal ballooning was found to be associated with VL, with the best cutoff for prediction being 26 cm^2^. Our results strengthen this finding, as we have also witnessed an increased levator hiatal area in patients with VL. Consistent with previously published data, women who complained of VL were younger [4]. This could be explained by the likelihood that younger women are more aware of and bothered by this symptom. Fewer menopausal women were found in the VL-positive group, which could be attributed to loss of elasticity and vaginal atrophy that might affect the feeling of a loose vagina.

In our study we aimed to better understand the pathogenesis of VL, to be able to provide women with better information and treatment. Regarding VL and POP symptoms, our results showed that women in the VL laxity group had more POP symptoms. This is not surprising given that the causes and risk factors seem to be similar. However, Alexander et al. [14], in their study, concluded that VL is not an early symptom of prolapse. Moreover, Polland et al. showed that VL measured by the Vaginal Laxity Questionnaire did not correlate with the examination findings of prolapse [15].

A major strength of this study is its relatively large population size and being, to the best of our knowledge, the first cohort trying to address the relationship between different delivery modes and VL. A major limitation of the study is its retrospective design and the fact that the data regarding mode of delivery were based on patient-reported information, implying the risk of recall bias. We did not have access to obstetric records; hence, we have no information on variables such as the duration of the second stage of labor and perineal tears. Finally, the study included exclusively urogynecology patients of mostly white ethnicity, which limits the generalizability of our findings.

In conclusion, vaginal laxity was found to be more prevalent in vaginally parous women. This effect seems to be largely attributable to the first delivery. Instrumental delivery seems to further increase the strength of this association.

## Data Availability

Data available on request from the authors.
